# Quantitative Analysis of Antibody Survival across the Infant Digestive Tract Using Mass Spectrometry with Parallel Reaction Monitoring

**DOI:** 10.3390/foods9060759

**Published:** 2020-06-09

**Authors:** Bum Jin Kim, Jiraporn Lueangsakulthai, Baidya Nath P. Sah, Brian Scottoline, David C. Dallas

**Affiliations:** 1 Nutrition Program, School of Biological and Population Health Sciences, College of Public Health and Human Sciences, Oregon State University, Corvallis, OR 97331, USA; bumjin.kim@oregonstate.edu (B.J.K.); lueangsj@oregonstate.edu (J.L.); baidya.sah@oregonstate.edu (B.N.P.S.); 2 Department of Pediatrics, Oregon Health & Science University, Portland, OR 97239, USA; scottoli@ohsu.edu

**Keywords:** parallel reaction monitoring, polyethylene glycol-28, palivizumab, nano-liquid chromatography/Orbitrap mass spectrometry, infant digestion, human milk, gastrointestinal contents

## Abstract

Orally delivered antibodies may be useful for the prevention of enteric pathogen infection, but to be effective they need to survive intact across digestion through the gastrointestinal tract. As a test case, we fed a recombinant human antibody, palivizumab, spiked into human milk to four infants and collected gastric, intestinal and stool samples. We identified a tryptic peptide from palivizumab (LLIYDTSK) that differs from all endogenous human antibodies and used this for quantitation of the intact palivizumab. To account for dilution by digestive fluids, we co-fed a non-digestible, non-absorbable molecule-polyethylene glycol 28-quantified it in each sample and used this value to normalize the observed palivizumab concentration. The palivizumab peptide, a stable isotope-labeled synthetic peptide and polyethylene glycol 28 were quantified via a highly sensitive and selective parallel-reaction monitoring approach using nano-liquid chromatography/Orbitrap mass spectrometry. On average, the survival of intact palivizumab from the feed to the stomach, upper small intestine and stool were 88.4%, 30.0% and 5.2%, respectively. This approach allowed clear determination of the extent to which palivizumab was degraded within the infant digestive tract. This method can be applied with some modifications to study the digestion of any protein.

## 1. Introduction

Therapeutic antibodies (immunoglobulins) have become an important and highly efficacious part of the therapeutic arsenal available for the treatment and prevention of a wide range of diseases, including viral and bacterial infections, autoimmunity, inflammation and cancer [1,2,3]. Almost all approved and marketed antibodies are administered parenterally to be dispersed systemically. Oral administration of therapeutic antibodies is less common, in part because such antibodies would need to survive intact against the harsh conditions of the gastrointestinal tract [4].

Several studies indicate that some orally administered antibodies can retain activity in the gastrointestinal tract. For instance, camelid serum-derived antibodies administered orally in neonatal mice prevented rotavirus infection and reduced rotavirus-associated diarrhea [5,6]. The oral administration of human serum immunoglobulin to low-birth-weight infants who were infected with rotavirus and experiencing chronic diarrhea eliminated the rotavirus infection [7]. Orally ingested bovine milk immunoglobulin concentrate reduced the duration and frequency of diarrhea induced by rotavirus and *Escherichia coli* in infants and children [8,9,10]. Orally administered doxorubicin monoclonal antibodies (MAD 11) reduced the gastrointestinal toxicity of this chemotherapeutic drug in mice [11].

To maintain therapeutic efficacy in the gastrointestinal tract after oral administration, the antibody’s structure must remain structurally intact against pH changes and proteolytic digestion within the digestive system [12,13]. Several studies have revealed that the amount of orally ingested immunoglobulins that survive across the digestive system is higher in infants than in adults, likely in part due to the infant’s higher gastric pH and lower levels of proteolysis in the gastrointestinal tract [14,15,16]. Therefore, use of orally delivered antibodies may be particularly relevant for infants.

To investigate the survival of antibodies in the infant digestive system, previous studies have relied on measuring the recovery rate of antibodies in stool samples [14,17]. Stool samples provide limited insight, as low antibody recovery in the stool does not necessarily reflect potential for that antibody to prevent infection. Antibody survival in the stomach and upper intestine could allow important pathogen inactivation and anti-adhesive activity, regardless of whether the antibody persists through the colon where it is exposed to bacterial proteases. To reliably evaluate the potential for orally administered antibodies to act within the digestive system, an approach to collect gastric and intestinal samples and quantify intact antibody survival is needed.

We have established a technique to sample the digestive contents from the infant stomach and small intestine via naso-gastric and post-pyloric (distal duodenal or proximal jejunal) tubes [18]. With this technique, we were able to collect these digestive samples from infants after feeding milk with added antibodies. After feeding the antibody and collecting digestive samples, a method is required for quantitation of the remaining intact antibody at each stage of digestion.

Proteins in complex biological samples can be quantified using liquid chromatography (LC) mass spectrometry (MS). Proteins in a sample can be digested by trypsin and a specific tryptic peptide can be separated by LC and quantified by MS in comparison with a standard curve. The selected tryptic peptide serves as a surrogate for the intact protein as a whole, thus allowing the quantification of the intact protein. Parallel-reaction monitoring (PRM) based on high-resolution MS platforms is an emerging technique for the quantification of target proteins in complex biological samples [19]. Orbitrap-based PRM monitors all product ions in the full MS/MS scan with high resolution [20], and, therefore, reduces the effort and time involved in creating the acquisition method [21].

Although quantitative analysis using LC/MS/MS enables the reliable quantification of the target proteins in complex biological samples, monitoring the survival of intact proteins across digestion includes additional analytical challenges. During digestion, a variety of fluids are secreted into the gastrointestinal tract and water is absorbed. Dilution of the sample would reduce the target protein concentration regardless of any digestive breakdown. To disambiguate between target degradation and dilution/concentration, we co-administered polyethylene glycol (PEG), a non-digestible, non-absorbable molecular marker, with the target antibody. We quantified PEG and used it to normalize the target protein concentration. PEG is widely employed in the treatment of children diagnosed with functional constipation (at much higher doses than used herein) and is recognized as safe by the United States Food and Drug Administration (FDA) [22]. PEG is neither absorbed nor digested within the human gastrointestinal tracts [23]. Concentrations of the antibody in digestive samples were normalized for dilution based on the ratio of original and measured concentrations of PEG to determine the actual degradation rate of the antibody across infant digestion.

Another issue in monitoring the intact survival of an antibody across infant digestion is that human milk contains an array of antibodies and infants can express and secrete antibodies into the gastrointestinal tract [24,25,26]. To track the antibody of interest, a means by which to differentiate between the target antibody and these endogenous antibodies is required. In this study, we administered palivizumab (Synagis^®^), an anti-respiratory syncytial virus humanized recombinant IgG1 monoclonal antibody approved by the FDA for intramuscular injection in infants [27]. Palivizumab contains 5% murine sequences and 95% human sequences [28,29,30]. The sequence of the variable (antigen-binding) region of palivizumab is clearly distinguishable from the sequences present in naturally occurring human milk and infant-produced antibodies. The uniqueness of the palivizumab variable region sequence enables the designation of surrogate tryptic peptides from this region that allow highly specific measurement without overlap from co-digested peptides derived from other proteins in quantitative analysis using LC/MS/MS.

In this study, we developed a method to determine the degree of intact survival of an antibody across the infant digestive tract with correction for dilution or concentration using oral administration of a recombinant antibody standard, palivizumab, in human milk, with the co-administration of a novel internal standard, PEG-28, sampling of the infant digestive tract and PRM-based nano LC/Orbitrap MS quantitation. This method was validated in four infants.

## 2. Materials and Methods

### 2.1. Sample Collection

All human milk samples and infant gastric, intestinal and stool samples were collected from the Doernbecher Children’s Hospital Neonatal Intensive Care Unit (NICU) located at Oregon Health & Science University in Portland, OR. This study was approved by the Institutional Review Board of Oregon Health & Sciences University (OHSU IRB 18274). The use of palivizumab for oral supplementation to neonates was given a “may proceed” clearance by the United States Food and Drug Administration (IND #140999).

Infants (*n* = 4) were selected for collecting samples based on the following criteria: infants already admitted to the NICU, greater than 34 weeks corrected gestational age, an indwelling nasogastric or orogastric feeding tube, and tolerating goal enteral feeding volumes (typically 150–160 mL/kg/day). Infants were excluded from the study if they had anatomic or functional gastrointestinal disorders that would impact protein digestion, were medically unstable or had been diagnosed with a condition that was ultimately non-viable (e.g., severe pulmonary hypoplasia, lethal trisomy). None of the infants in the NICU were receiving PEG-3350 during the course of the study.

After obtaining informed consent from parents, a 5 Fr soft, weighted feeding tube was placed into the distal duodenum or proximal jejunum through a naris by a NICU by a nurse experienced in such placement without need for anesthesia. The position of the tube port was confirmed by abdominal X-ray. The tube was in place for two days for the purposes of the palivizumab survival studies. Human milk was mixed with palivizumab and PEG-28 and fed via either the naso- or oro-gastric tube, or by bottle feeding over 30 min or less. A 2-mL sample of the feed with palivizumab and PEG-28 supplementation was collected. The gastric contents (0.5–2 mL) were collected by syringe suction from each infant between 30 min and 1 h after initiation of feeding and placed into sterile vials on ice. Intestinal samples were collected from the nasojejunal/duodenal tube via gravity flow as the digesta passed the collection tube port into sterile vials on ice. Multiple stool samples were collected from diapers into sterile containers within the first 24 h after a feed. After collection, all samples were immediately stored at −80 °C. For analysis, all samples were transported to Oregon State University on dry ice and stored at −80 °C upon arrival. Sample information is shown in Table S1.

### 2.2. Preparation of Human Milk, Infant Digestive Samples and the Standard Mixture

All samples were centrifuged at 4000× *g* at 4 °C for 30 min and the infranatant (between the upper lipid layer and lower pellet), which included palivizumab and PEG-28, was collected into a new tube. Stool samples (0.1 g) were dissolved with 1 mL of 0.05% Tween-20 (Bio-Rad, Richmond, CA, USA) in phosphate-buffered saline (PBS; pH 7.4; Thermo Scientific, Waltham, MA, USA) solution (PBST) prior to centrifugation. These samples were re-centrifuged with the same conditions and the infranatant was re-collected in another new tube. Samples were frozen at −80 °C until the next sample preparation step. To make calibration curves for palivizumab peptide and PEG-28, 10 μL of 10 μg/μL commercial palivizumab standard (MedImmune, Gaithersburg, MD, USA) and 10 μL of 100 ng/μL PEG-28 (Sigma Aldrich, St. Louis, MO, USA) were mixed with 10 μL of nanopure water and used as a standard mixture.

In parallel, human milk samples spiked with a known amount of palivizumab were prepared to test the PRM-MS-based quantitation method and evaluate the recovery and reproducibility of the method. Eighty-eight microliters of human milk samples (*n* = 2) were mixed with 10 µL of 1 µg/µL palivizumab and 2 µL of 10 ng/µL PEG-28. The same sample preparation procedure applied for the clinical human milk and infant digestive samples was carried out for these spiked samples.

### 2.3. Separation of Intact Palivizumab Proteins from Endogenous Protease-Produced Peptides and PEG-28

To separate intact palivizumab proteins from its peptides digested by proteases in human milk and gastrointestinal fluids, ethanol (EtOH) precipitation was performed for all samples and the standard mixture. Thirty microliters of samples and the standard were mixed with 150 μL of chilled (−20 °C) ethanol (Sigma Aldrich, St. Louis, MO, USA) and stored at −20 °C for 1 h. Samples were centrifuged at 12,000× *g* at 4 °C for 20 min to precipitate the proteins. The supernatant, including endogenous peptides and PEG-28, was collected and dried by vacuum centrifugation.

### 2.4. Trypsin Digestion of Palivizumab

For both samples and the standard mixture, the EtOH-precipitated pellets, which contained the intact palivizumab, were mixed with 100 μL of 50 mM ammonium bicarbonate (Thermo Scientific, Waltham, MA, USA), followed by the addition of 2 μL of 550 mM dithiothreitol (4 μL for the standard) (Promega, Madison, WI, USA) and incubated at 50 °C for 50 min to reduce disulfide bonds. After incubation, 4 μL of 450 mM iodoacetamide (8 μL for the standard) (Sigma Aldrich, St. Louis, MO, USA) was added and incubated at room temperature (RT, 20–23 °C) for 1 h in the dark to alkylate the thiol groups. To digest the denatured proteins into peptides, 2 μL of 1 μg/μL trypsin (4 μL for the standard) (Promega, Madison, WI, USA) was added and mixtures were incubated at 37 °C with shaking at 300 rpm overnight.

A stable isotope-labeled synthetic (SIS) peptide with ^13^C_6_ and ^15^N_2_ at the lysine (K) of LLIYDTSK (New England Peptide, Gardner, MA, USA) was spiked into each sample (3 μL of 10 ng/μL SIS peptide) and standard (10 μL of 100 ng/μL SIS peptide) and these tryptic peptides were combined with the supernatant from the EtOH precipitation step for each sample.

### 2.5. Enrichment and Purification of Palivizumab-Derived Tryptic Peptides and PEG-28

Tryptic peptides of palivizumab and PEG-28 in samples and the standard mixture were purified by solid-phase extraction with C18 cartridges (Sigma Aldrich, St. Louis, MO, USA). Cartridges were washed with 5 mL of 80% acetonitrile (ACN; Merck, Darmstadt, Germany), 0.1% trifluoroacetic acid (TFA; Merck, Darmstadt, Germany) in nanopure water and reconditioned with 5 mL of nanopure water prior to loading the samples, which included the tryptic palivizumab peptides, the SIS peptide and PEG-28. After sample loading, the cartridges were washed with 5 mL of nanopure water to remove salts and interfering substances. Five milliliters of 80% ACN, 0.1% TFA in nanopure water were added to elute peptides and PEG-28. All samples and the standard mixture were dried by centrifugal evaporation at RT. Samples and the standard mixture were reconstituted with 30 μL and 100 μL of 3% ACN, 0.1% formic acid (Merck, Darmstadt, Germany) in nanopure water, respectively. To build the calibration curve, the standard mixture was diluted with the reconstitution solvent at 20,000×, 10,000×, 4000×, 2000×, 1000×, 400×, 200× and 100×. The concentrations of the standard series used were 0.5, 1, 2, 5, 10 and 20 ng/μL for palivizumab; 0.01, 0.05, 0.1, 0.2, 0.5 and 1 ng/μL for the SIS peptide; and 0.005, 0.01, 0.05, 0.1, 0.5 and 1 ng/μL for PEG-28.

### 2.6. Data-Dependent Analysis-Based Overall Characterization of Tryptic Palivizumab Peptides

Tryptic palivizumab peptides were analyzed using a Waters nanoACQUITY UPLC (Waters, Milford, MA, USA) with an Orbitrap Fusion™ Lumos™ Tribrid™ mass spectrometer (Thermo Scientific, Waltham, MA, USA). Ten microliters of the reconstituted sample were diluted with 10 μL of 3% ACN, 0.1% formic acid in nanopure water. One microliter of each sample was loaded onto a C18 180 μm × 20 mm, 5-μm bead nanoAcquity UPLC trap column (Waters, Milford, MA, USA) for enrichment and desalting, and separated with a 100 μm × 100 mm, 1.7 μm bead Acquity UPLC Peptide BEH C18 column (Waters, Milford, MA, USA) over 60 min.

All peptides were separated at a flow rate of 0.5 µL/min with a gradient elution using solvent A (100% nanopure water with 0.1% formic acid) and solvent B (100% ACN with 0.1% formic acid): 3% to 11.5% B, 0 min to 10 min; 11.5% to 20% B, 10 min to 31 min; 20% to 30% B, 32 min to 36 min; 30% to 95% B, 36 min to 45 min; 95% B, 45 min to 54.5 min, 95−3% B over 0.5 min, and finally the column was re-equilibrated with 97% A for 5 min.

Molecules in the samples were ionized with an electrospray voltage of 2350 V and the ion transfer tube temperature was 300 °C. Full scan MS spectra were acquired in positive ionization mode over an m/z range of 200–2000 with a resolution of 60,000. The automatic gain control target was set to 4.0 × 10^5^, with a maximum injection time of 50 ms. The MS cycle time was set to 3 s. Following an MS scan, precursor compounds were automatically selected for MS/MS analysis by the acquisition of software based on the following criteria: ion-intensity threshold 5.0 × 10^4^, charge state 2–8 and exclusion time 60 s. The precursor ions selected were fragmented using higher-energy collisional dissociation (HCD; normalized collision energy was set to 30%). All MS/MS spectra were acquired in the positive ionization mode by the Orbitrap at resolution of 30,000. The automatic gain control (AGC) target was set to 5.0 × 10^4^.

### 2.7. Determination of the Unique Palivizumab

Tryptic palivizumab peptides were identified from the MS raw files using Thermo Proteome Discoverer (v2.2), and a SequestHT search engine based on the protein sequence database containing human IgG 1, 2, 3 and 4 proteins (heavy constants) and the variable regions of the heavy (VH) and light (VL) chains of palivizumab. The cleavage sites were set to C-terminal arginine (R) and lysine (K) and digestion specificity was selected as fully specific with a maximum of two missed cleavages. The precursor mass tolerance was set to 10 ppm with fragment mass tolerance of 0.1 Da. Potential modifications included phosphorylation of serine and threonine and oxidation of methionine. Carbamidomethylation of cysteine was specified as a fixed modification. The abundances of the peptides were determined by measuring the area under the curve of the eluted chromatogram peak.

To determine a specific unique palivizumab peptide for quantitation, the sequences, modification and frequency of peptides derived from the palivizumab VL and VH regions were investigated based on the data-dependent analysis-based MS/MS results. The uniqueness of all peptides identified were evaluated by the peptide uniqueness checker in neXtProt (http://www.nextprot.org/).

### 2.8. PRM-Based Quantitation

The columns and LC/MS acquisition parameters used for the PRM-based quantification matched those described above for the data-dependent analysis with the following exceptions. Tryptic peptides, the SIS peptide and PEG-28 were separated and eluted using the following 20 min LC gradient conditions: 3% to 13% B, 0 min to 5.5 min; 13% to 20% B, 5.5 min to 11.5 min; 20% to 30% B, 11.5 min to 13 min; 30% to 95% B, 13 min to 14.5 min; 95% B, 14.5 min to 16.5 min; 95−3% B over 0.5 min, and then the column was re-equilibrated with 97% A for 3 min. The unique palivizumab peptide at m/z 476.7711 (z = 2), the SIS peptide at m/z 480.7782 (z = 2) and PEG-28 at m/z 417.933 (z = 3) were selected as targeted precursor compounds for fragmentation. The peptides were fragmented using HCD (0–18 min) and PEG-28 was fragmented using collision-induced dissociation (CID; 18–20 min) with 35% of normalized collision energy. MS and MS/MS spectra were acquired by the Orbitrap at a resolution of 60,000. The MS/MS scan ranges were m/z 200–1500 (for peptides) and m/z 300–1500 (for PEG-28).

### 2.9. Data Processing for Quantitation and Statistical Analysis

Data processing, including making a standard curve and calculating the concentration of the unique peptide from palivizumab, the SIS peptide and PEG-28 in standards and samples, was performed using Skyline (MacCoss Lab, University of Washington, WA, USA) [31]. The MS/MS raw data sets were imported into Skyline and the peak areas were extracted using the selected transition list for each precursor ion. Transitions were extracted from raw files using the following parameters in Skyline: m/z 0.01 in the method match tolerance; data independent analysis in the acquisition method; Orbitrap in the product mass analyzer; all ions in the isolation scheme; 60,000 at m/z 200 in the resolving power for PEG-28 and m/z 0.01 in the method match tolerance; targeted in the acquisition method; Orbitrap in the product mass analyzer; 60,000 at m/z 200 in the resolving power for palivizumab peptides.

Concentrations of peptides and PEG-28 were calculated based on each individual calibration curve. The palivizumab concentration in each sample measured via PRM was normalized based on comparing actual and measured concentrations of SIS peptide and PEG-28 present in the sample. The recovery rate of the spiked palivizumab in the human milk sample was calculated by the following equation: measured concentration ÷ spiked actual concentration × 100. The limit of detection and limit of quantitation were determined as the analyte concentration corresponding to the sample blank value (mean concentration of the blank) plus three standard deviation and ten standard deviations, respectively. Palivizumab concentrations after adjustment by the SIS peptide and PEG-28 were compared between the feed and digestive samples using paired Student’s *t*-tests.

### 2.10. Anti-Idiotype Palivizumab Enzyme-Linked Immunosorbent Assay (ELISA)

All ELISAs for palivizumab were performed according to the methods described by Bio-Rad with some modifications. Briefly, the anti-palivizumab antibody HCA261 (Bio-Rad, Richmond, CA, USA) at 1 μg/mL in 1 × PBS (PBS 10×, pH 7.4, Thermo Scientific, Waltham, MA, USA) was coated onto clear flat-bottom Immuno 96-well plates MaxiSorp (Thermo Scientific, Waltham, MA, USA) with 100 μL per well and incubated overnight at 4 °C. After incubation, the microtiter plate was washed three times with 200 μL of PBST using a Wellwash™ Versa microplate washer (Thermo Scientific, Waltham, MA, USA) and blocked for 1 h with 150 μL of 5% of bovine serum albumin (Blocker™ BSA (10×) in PBS, Thermo Scientific, Waltham, MA, USA) diluted with PBST at RT. After washing three times, the standards and samples were added to the wells (100 μL) and incubated for 1 h at RT. The standards were prepared using palivizumab (Synagis, MedImmune, Gaithersburg, MD, USA) in serial dilutions (from 0–10,000 ng/mL) in PBST with 10% human AB serum (Corning, Manassas, VA, USA). The samples were diluted with 10% of human AB serum in PBST at 200× and 400× for milk, gastric and intestinal samples, and 1× and 2× for stool samples. The samples were added in wells (100 μL) and incubated at RT for 1 h. After incubation and washing, horseradish peroxidase-conjugated detection antibody HCA262P (Bio-Rad, Richmond, CA, USA) was diluted in HISPEC immunoassay diluent (Bio-Rad, Richmond, CA, USA) at 2 μg/mL, added to wells (100 μL) and incubated at RT for 1 h. The plates were washed three times with PBST and the substrate (1×, 100 μL), 3,3′,5,5′-tetramethylbenzidine (Invitrogen, San Diego, CA, USA), was added into wells and incubated for 5 min at RT followed by addition of 50 μL of 2N sulfuric acid to stop the coloration reaction. The optical density was measured at 450 nm with a spectrophotometer (SpectraMax M2, Molecular Devices). Data were interpreted using four-parameter logistic models to make the standard curve on each plate with R^2^ > 0.99 for goodness fit using Softmax^®^ Pro 7.0 software.

## 3. Results and Discussion

### 3.1. Overall Workflow for Absolute Quantitation Using PRM-MS

**Figure 1:**
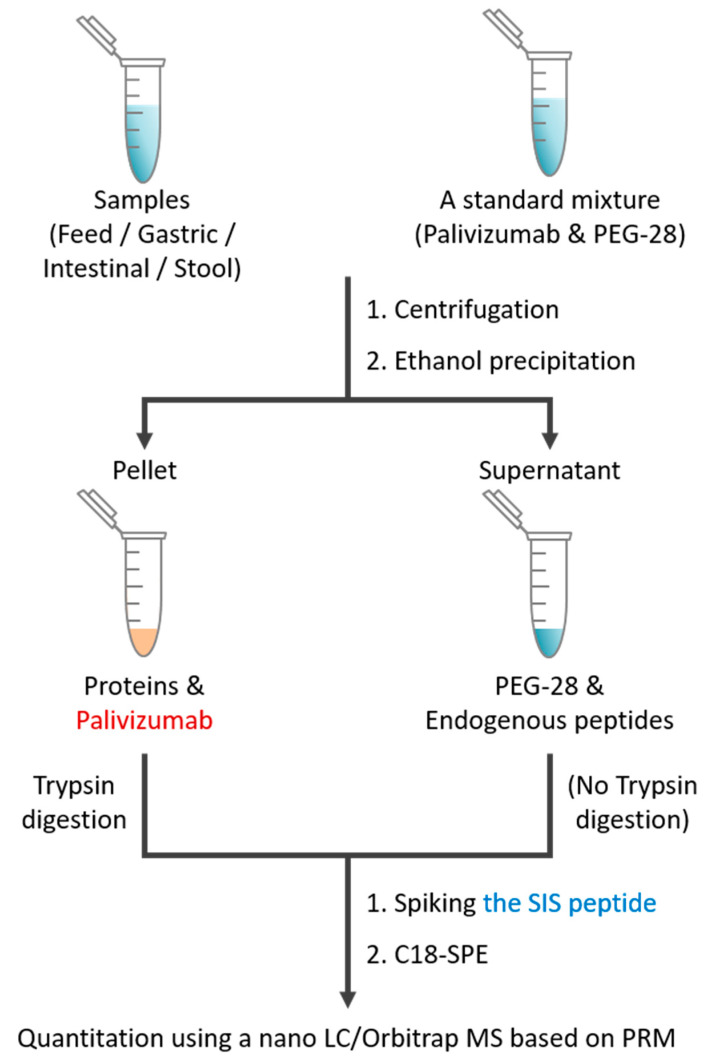
Experimental workflow for determining the concentration of palivizumab and PEG-28 in feed and digestive samples by PRM-based LC/MS analysis.

The experimental procedure for the quantitation of palivizumab and PEG-28 in human milk and digestive samples by PRM-based MS analysis are presented in Figure 1. Proteins (including palivizumab) enriched by centrifugation and EtOH precipitation were digested by trypsin in order to make a surrogate peptide of the parent protein for PRM-based quantitation of the target protein. After proteolytic digestion, a synthetic stable isotope-labeled version of the unique palivizumab-derived peptide was immediately spiked in the samples to later correct for subsequent extraction and analytical variation of the unique palivizumab peptide. These samples were then mixed with the EtOH supernatant (which contained the PEG-28) from each original sample to allow simultaneous analysis of both peptides and PEG-28 in a single MS/MS run. In the EtOH supernatant, no unique palivizumab peptide selected herein was found via full MS/MS scanning, whereas PEG-28 was detected in abundance (Figure S1). This finding indicates that the unique palivizumab peptide measured in the combined tryptic digest of the protein pellet and undigested supernatant derived solely from the EtOH-precipitated trypsin-digested intact palivizumab, and not from palivizumab peptides released by human milk or gastrointestinal system proteases. PEG-28 is separated from proteins by the EtOH precipitation and is present in the EtOH supernatant. Samples were purified by C18-solid phase extraction and analyzed using PRM-based a nano LC/Orbitrap MS.

### 3.2. Determination of the Unique Peptide of Palivizumab for PRM

The selection of the representative tryptic peptides present only in the target protein is essential for the PRM-based quantitation of a specific protein in a sample [32,33,34]. Palivizumab is a humanized IgG and its constant regions of heavy and light chains are derived from human IgG1 while its variable regions (VL and VH) are humanized based on the murine sequences derived from a murine monoclonal antibody (Mab 1129) [35]. The unique peptide would need to be able to differentiate between palivizumab and the natural IgG present in the samples derived from milk and potentially from infant secretions. As the constant region of palivizumab is derived from human IgG, we selected unique peptides from the variable regions (VL and VH) of palivizumab.

All tryptic peptides from the commercial palivizumab standard were characterized using data-dependent analysis-based full scanning using a nano LC/Orbitrap MS. The tryptic peptides found from commercial palivizumab standards ranging from 2 to 25 ng/µL are listed in Table 1. Of the ten tryptic peptides from the known palivizumab VL and VH regions, only two peptides, LLIYDTSK and VTNMDPADTATYYCAR (with carbamidomethylation), were detected across the entire concentration range. Next, we identified whether the peptide sequences observed in MS analysis were unique using the peptide uniqueness checker in neXtProt [36]. This analysis revealed that seven out of ten peptides did not match any sequences in the human protein database. Both LLIYDTSK and VTNMDPADTATYYCAR (the two peptides detected across all concentration ranges of standards) were among the unique peptides. These two peptides did not match any peptide sequences of any human protein, including native human IgG.

**Figure 2:**
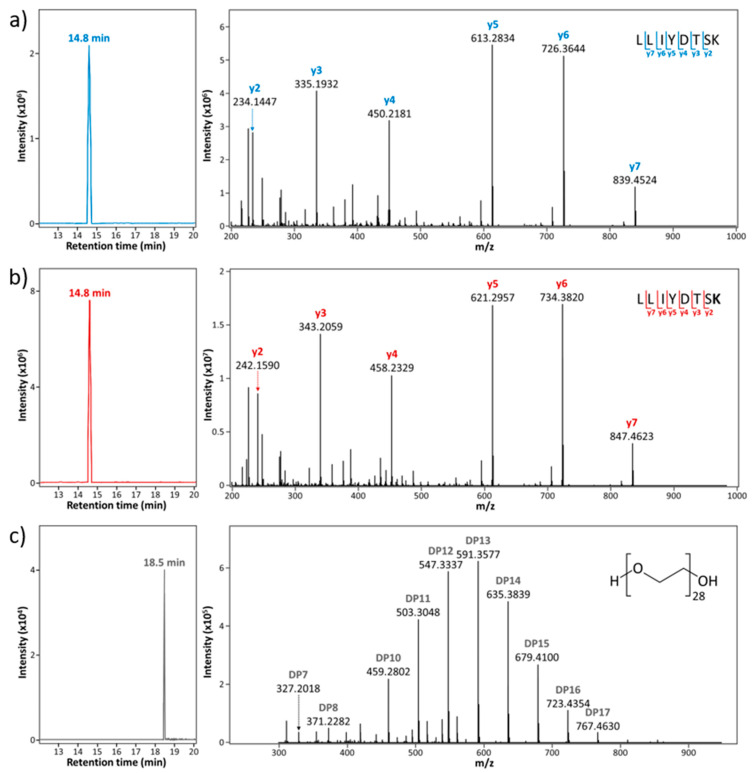
Extracted ion chromatograms (left) and tandem MS spectra (right) of the unique peptide of palivizumab, the SIS peptide and PEG-28 in the standard mixture: (**a**) the unique peptide at m/z 476.771 (z = 2, 14.8 min); (**b**) the SIS peptide at m/z 480.778 (z = 2, 14.8 min); and (**c**) PEG-28 at m/z 417.922 (z = 3, 18.5 min).

As the carbamidomethylation of VTNMDPADTATYYCAR may not occur in all instances of the peptide, we did not select this peptide for quantification. LLIYDTSK was unmodified, fully digested by trypsin (no missed cleavage site) and was distinctly separated with the internal standard, PEG-28, on the LC chromatogram (refer to chromatograms in Figure 2a,c). The chromatogram peak abundances of LLIYDTSK were higher than VTNMDPADTATYYCAR. Therefore, we selected LLIYDTSK as the representative unique palivizumab-derived peptide for the quantitation of intact palivizumab.

### 3.3. Selection of Transitions of the Palivizumab Peptides and PEG-28

Among approaches for targeted quantitation using LC/MS/MS, single and multiple reaction monitoring (SRM and MRM) are common. Within this approach, many surrogate peptides (and thus, target proteins) can be simultaneously quantified within a single analysis [37,38]. PRM increases specificity by including more transition ions compared with SRM and MRM and measuring them with higher resolution, thereby reducing interference from co-isolated background ions for target identification compared with SRM and MRM. In the Orbitrap MS-based PRM, a precursor ion is selected for isolation in the quadrupole and its fragment ions are produced in the fragment cell. Unlike SRM and MRM, PRM does not require the pre-selection of fragment ions at MS/MS analysis as it uses full MS/MS scanning to measure all fragment ions of each target precursor. To determine the fragment ions derived from the palivizumab peptides and PEG-28 in each MS/MS spectrum, a list of potential transitions (pairs of a precursor ion and fragment ions) was generated based on the annotation of fragment ions in the tandem MS spectra.

We first examined the chromatographic elution patterns for each to identify the retention times of the peptides of interest and PEG-28. The selected unique palivizumab peptide (LLIYDTSK) and the matching stable isotope-labeled standard peptide (modified with ^13^C_6_ and ^15^N_2_ at lysine (K)) were clearly separated from PEG-28 by C18 column-based LC separation. As shown in the extracted ion chromatograms in Figure 2, the SIS peptide co-eluted (14.4 min) with the unique peptide, which is as expected as the stable isotope replacement does not alter the peptide’s physicochemical interactions with the column. PEG-28 eluted at 18.5 min. This distinct separation of LC retention time achieved from the current LC conditions ensured that PEG-28 and the peptides of interest did not impact each other’s ionization efficiency, allowing for an optimal sensitivity.

The fragment ions of the unique palivizumab peptide, the SIS peptide and PEG-28 that eluted at each specific LC elution time point were added to the transition lists for PRM-based quantitation. Doubly charged ions of the unique palivizumab-derived peptide (m/z 476.771) and the stable isotope-labeled peptide (m/z 480.778) were selected as the precursor ions and their product ions were acquired after HCD-fragmentation (Figure 2a,b). The annotated MS/MS spectra of the unique and the SIS peptides had mainly y-type fragment ions of each precursor ion in abundance. As all y-ions included the C-terminal lysine, which was labeled for the SIS peptide, these ions all differed by 4 m/z (8 Da) between the two peptides. Thus, we included only the y-ion series (y2–7) in the transition list to ensure specific quantitation of the labeled and unlabeled form of the peptide (Table 2).

For the PRM of PEG-28, triply charged PEG-28 (m/z 417.922) was chosen as the precursor ion and fragmented by CID (Figure 2c). In the tandem MS spectrum of PEG-28, ten product ions with degree of polymerization (DP) 7 to 17 (except DP9) were in abundance and selected for PRM-based quantitation. We examined the use of both CID and HCD fragmentation for PEG-28. HCD fragmentation resulted in spectra dominated by one abundant peak (DP5-18 Da) with low abundances of eight other fragment ions (DP5 to 11) (Figure S2). CID fragmentation resulted in tandem MS spectra containing clear diagnostic peaks that covered a wide DP range. The more balanced fragmentation spectra after CID allowed for more confident quantification of PEG-28. Consequently, we selected CID for PEG-28 fragmentation and used a transition list generated from CID-fragmentation for PRM.

Prior to selecting PEG-28 as the internal standard to account for dilution/concentration, we had planned to use PEG 3350. The analysis of PEG 3350 by LC/MS, however, revealed a highly complex array of PEG polymers that eluted at the same retention time (Figure S3). These PEG polymers were composed of various numbers of repeated ethylene oxide (O–CH_2_–CH_2_) units with each PEG polymer differing by 44 Da (1 unit of ethylene oxide), each of which appearing in numerous charge states. Because of the numerous masses represented at many charge states, the isolation of a single precursor of interest based on m/z value is difficult. Without complete isolation, quantification based on PRM would be inaccurate because the PEG polymers produce the same array of fragment ions after collision-induced dissociation (fragmenting at the carbon-oxygen bonds between ethylene oxide units), preventing fragment ion-based disambiguation. For this reason, we selected a single molecule, PEG-28, as the internal standard rather than PEG 3350.

### 3.4. PRM-Based Quantitation of Palivizumab and PEG-28 in Human Milk

We performed the PRM-based quantitative analysis using a human milk sample spiked with a known amount of palivizumab. Two milk samples were prepared in independent experiments. Each sample was measured four times by PRM-MS. This design enabled us to evaluate the reproducibility of the experimental procedures and the instrument.

**Figure 3:**
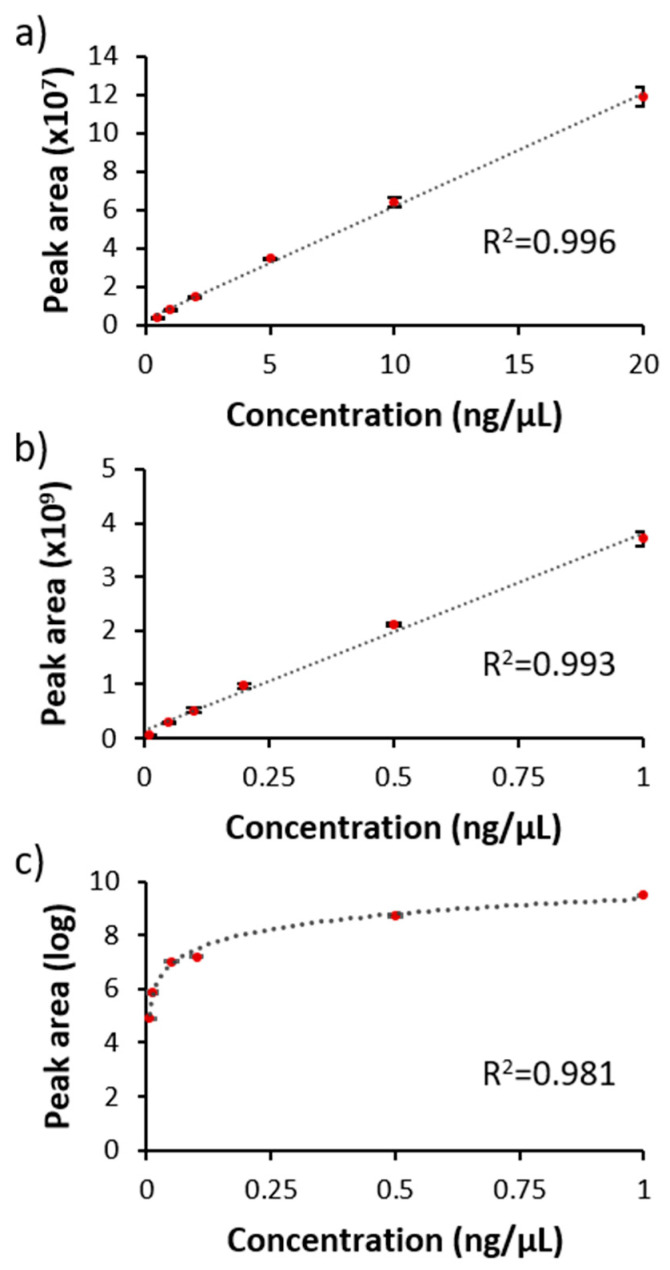
Calibration curves of: (**a**) the unique peptide (LLIYDTSK) of palivizumab; (**b**) the SIS peptide; and (**c**) PEG-28. The error bars represent standard deviations of three measurements.

Calibration curves across a series of dilutions of the standard mixture were built for the unique peptide, the SIS peptide and PEG-28 (Figure 3) based on each molecule’s specific transition list (Table 2). The calibration curves of the palivizumab unique peptide (LLIYDTSK) and SIS peptide were fitted linearly with *R^2^* = 0.996 and 0.993. The PEG-28 standard series fit with a logarithmic calibration curve (*R^2^* = 0.981). The limits of detection of the palivizumab unique peptide, the SIS peptide and PEG-28 were 45.20, 0.44 and 0.45 pg/µL, respectively, and their limits of quantitation were 46.08, 0.46 and 1.32 pg/µL, respectively.

The concentration of the SIS peptide and PEG-28 in the spiked human milk samples were measured using these calibration curves. The expected concentration of the SIS peptide in 1 µL of human milk sample injected in LC/MS was 0.5 ng/µL, and the average of all measured values was 0.39 ng/µL, indicating an average recovery after C18 SPE of 77.6%. Palivizumab was spiked in human milk samples at 50 ng/µL and the average of all measured values was 15.16 ng/µL. To correct the experimental and instrumental variations of the unique palivizumab peptide, all measured values of palivizumab were normalized using the average measured concentration of SIS peptide. The measured average concentration of palivizumab was normalized to 19.43 ng/µL.

The recovery rate of palivizumab based on the normalized concentration was 38.9%. The measured concentrations of palivizumab in the four feeds (palivizumab spiked in the clinic) had similar recovery rates: on average 32% recovery. PEG-28 (spiked in human milk at 0.1 ng/µL) was measured as 0.074 ng/µL, yielding a calculated recovery of 74.2% on average. The main reason for the incomplete recovery of both the unique palivizumab peptide and PEG-28 may be that the matrix of milk was much more complex than that of the standard, which could lead to loss in the sample preparation steps, particularly in solid phase extraction clean-up, and signal suppression in MS by other peptides and background molecules. Another possible reason for incomplete recovery is that endogenous human milk proteases could have degraded palivizumab to EtOH precipitation, resulting in a lower yield of the target peptide (LLIYDTSK).

Variations of instrumental and experimental replications were investigated based on the percent coefficient of variation (CV) of the measured concentration of each target molecule to evaluate the reproducibility of the LC/MS measurements and sample preparation. The precision of the four instrumental replicates was 8.1%, 10.3% and 5.5% for the unique peptide, SIS peptide and PEG-28 for the first sample, respectively. In the second sample, the precision of the four instrumental replicates of the unique and SIS peptides and PEG-28 was 4.9%, 4.4% and 2.1%, respectively. The experimental variations between the first and second spiked milk samples were 6.5%, 6.1% and 0.8% for the unique peptide, SIS peptide and PEG-28, respectively.

The concentrations measured by PRM-quantitation were highly reproducible in both repeated instrument measurements and experiments. These findings indicate that the low recovery of the palivizumab unique peptide was not due to variations of the instrument (nano LC/Orbitrap MS) and sample preparation.

### 3.5. Monitoring of Palivizumab Degradation in Digestive Samples

Using PRM-quantitation combined with an internal standard (PEG-28)-based normalization, we measured the concentrations of intact palivizumab in feed (human milk), gastric and intestinal fluid and stool samples to monitor how much palivizumab was degraded during digestion in the infant digestive tract. Human milk spiked with palivizumab and PEG-28 was fed to infants (*n* = 4) and gastric and intestinal fluids samples were collected 0.5–1 h and 0.5–1.5 h after the initiation of feeding, respectively, and stool samples were collected within 24 h of feeding. Sample and standard mixture preparation and PRM-based quantitation were performed by the experimental procedure, as described in the Materials and Methods section.

The palivizumab concentration measured through PRM-quantitation in feed and the digestive samples of each infant and their normalized values considering their dilution by digestive secretions or concentration by water absorption are shown in Table 3. Comparison of the palivizumab concentration in each sample revealed that palivizumab was continuously degraded as it passed from feed across the gastrointestinal tract. In particular, less than half of the original feed concentration of palivizumab was found in all the intestinal samples. Gastric proteases, namely pepsin, and pancreatic proteases, including trypsin, chymotrypsin, carboxypeptidase and elastases, secreted by the infant cause protein digestion, releasing peptides and amino acids [39,40]. These digestive enzymes could contribute to the observed palivizumab digestion across infant digestion. Milk proteases may continue to function across infant digestion [41,42] and also contribute to the observed palivizumab degradation. We detected either no, or only a minimal amount, of palivizumab unique peptide in the stool samples, suggesting that transit through the lower small intestine and colon led to complete degradation, likely via continued interaction with intestinal proteases and contributions from microbial proteases.

The dilution correction factors were calculated based on observed changes in PEG-28 concentration from the original feed across the digestive samples. All the dilution factors of the gastric and intestinal samples were more than 1, indicating that digestive fluids had been secreted, leading to the dilution of feed samples in all the digestive samples. All the dilution factors of intestinal samples were higher than those of the gastric samples. The continual reduction of PEG-28 concentration as milk transits the stomach and upper small intestine is due to continual dilution by secretion of gastrointestinal juice from the stomach and pancreatic juice in the small intestine [43]. The decrease in PEG-28 is not a result of degradation, as the infant lacks enzymes that can degrade this molecule and cannot absorb it. We observed variations among infants in the gastric and intestinal sample dilution factors. These variations may be because the individuals vary in the amount of digestive juices secreted. This observation is similar to the large range of gastric and duodenal juice secretion volumes observed in adults [44]. Therefore, the determination of the dilution factor for each infant is necessary for reliable measurement of the protein degradation across digestion. In the stool samples, we observed that two samples showed “dilution” and two showed “concentration.” We expected to observe only concentration. The observed dilution in two samples was likely due to differences in gastrointestinal transit times between infants, leading to variable timing of the bolus of PEG-28 arriving in the stool. Therefore, for the stool samples as collected in this protocol, PEG-28 did not serve as a marker of feed dilution or concentration but rather as an important factor to normalize the palivizumab concentration to account for these varying transit times.

**Figure 4:**
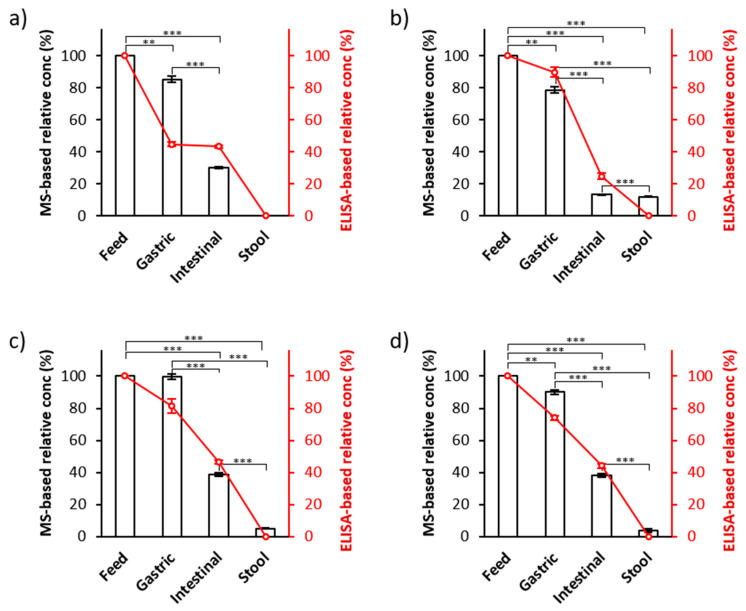
The relative quantitation of palivizumab in feed, gastric, intestinal and stool samples of: (**a**) infant 1; (**b**) infant 2; (**c**) infant 3; and (**d**) infant 4 measured by PRM-MS and ELISA. The bar graphs (white) and line charts (red) display the relative concentration of palivizumab as measured by PRM-MS and ELISA, respectively. The relative concentrations of intact palivizumab in gastric, intestinal and stool samples were normalized against the absolute concentration measured in each feed. *p*-Values of normalized concentrations of PRM-quantitation were calculated by paired Student’s *t*-test. * (one asterisk), ** and *** indicate *p* < 0.05, < 0.01 and < 0.001, respectively.

To ensure the efficacy of this MS-based palivizumab quantitation approach, we compared these MS-based results with the results from ELISA-based quantitation of the same samples (the line charts in Figure 4). We compared the percentage survival of palivizumab across infant digestion (calculated as the palivizumab concentration measured in the digestive sample divided by the palivizumab concentration in the feed samples × 100) between MS- and ELISA-based quantitation. On average, 88.4% (standard error ± 4.4%) and 72.4% (±9.8%) of palivizumab originally present in the feed was found in gastric samples by PRM- and ELISA-based quantitation, respectively. In the intestinal samples, 30.0% (±5.9%) and 39.7% (±5.0%) of the original feed palivizumab concentration were observed in PRM- and ELISA-based results, respectively. For stool samples, 5.2% (±2.5%) and 0.01% (±0.01%) of the feed palivizumab concentration were observed in the PRM- and ELISA-based results, respectively. PRM- and ELISA-based quantitation showed similar, consistent degradation rates across the infant digestive samples. The palivizumab concentrations in all intestinal and stool samples were statistically different from their respective feed and gastric samples (*p* < 0.001). Though we did not optimize stool sample collection time to obtain maximal palivizumab concentrations, co-fed and co-transiting PEG-28 normalization effectively controls for variance in palivizumab excretion across different fecal sample times.

These findings align with those of our previous studies of digestion via peptidomics, which indicated the continual release of peptides from human milk proteins over time in the infant stomach and, by implication, the continual degradation of intact milk proteins [45]. This finding also agrees with our previous work indicating that palivizumab is degraded by incubation in infant gastric and intestinal fluids (ex vivo digestion) [18]. The observed degradation of palivizumab was likely due to proteolytic degradation by pepsin in the stomach and by an array of intestinal proteases. The apparent loss of palivizumab is most probably due to proteolytic degradation rather than simple pH-induced denaturation, as all sample proteins were intentionally denatured during the EtOH precipitation step.

Though both ELISA and PRM-MS analysis showed a similar trend of palivizumab survival across the infant digestive tract, there were some variations in the measured concentrations between the two quantitation methods. The observed relatively larger difference between ELISA and PRM-MS measurements for the infant 1 gastric sample could be due to either sample preparation variance or instrument error during either the ELISA or PRM-MS analysis.

## 4. Conclusions

We have introduced a PRM-based quantitation method to monitor the extent of intact palivizumab degradation in infant digestion. Our results indicate that absolute quantitation using PRM-MS allows highly specific and sensitive measurement of palivizumab within complex biological samples that contain a large array of abundant, non-target proteins.

We demonstrated the feasibility of co-administering and quantifying a nondigestible, nonabsorbable internal standard (PEG-28) to normalize the palivizumab concentration for the dilution by gastrointestinal fluids. Controlling for dilution is essential because without this, the observed decreases in intact protein concentration across digestion could have been due to either digestion or dilution; thus, a control for dilution is required for disambiguation between these two possibilities. We have demonstrated that feeding a known amount of a nondigestible internal standard followed by PRM-based quantitation allowed normalization for dilution by digestive fluids across the digestive tract. This method enabled the reliable determination of the actual degradation rate of intact palivizumab during gastrointestinal digestion in the infant.

Although we established a method for the quantitative analysis of intact palivizumab across digestive samples and normalization of its measured concentration for dilution, there are limitations for applying this approach for quantifying the intact human milk proteins in the digestion samples. PRM-MS-based absolute quantification requires the selection of a unique surrogate peptide derived from the target protein. This requirement is problematic for the quantification of human milk proteins as many could potentially be produced by the infant and secreted into the digestive tract. This means that the unique surrogate peptide would not be unique to milk. Thus, observed “milk” protein concentration could derive from a combination of milk proteins and infant secreted proteins. An approach to overcoming this obstacle would be to label mother’s milk by feeding the mother stable isotopes and to follow the survival of the resulting isotope-labeled unique surrogate peptide across infant digestion. Further research is needed to determine the effectiveness of such an approach. The observation of bovine milk proteins would not require a labelling approach as protein sequence variation between bovine and human milk can be used to differentiate between the dietary and digestive secreted proteins.

The present study has shown a low recovery of intact palivizumab in human milk samples. Optimizing enrichment techniques for the target protein could increase the quantitation accuracy of the target protein. We validated this measurement technique with four infant samples. This approach can now be applied to larger sample sets to examine the extent of variation in digestion of palivizumab and other milk proteins among subjects. We will apply this approach to assess the survival of different isoforms (IgA and sIgA) of palivizumab.

Ultimately, the method created in this study can be used to quantify the intact survival of any recombinant antibody in the infant’s gastrointestinal tract to evaluate its potential efficacy for treatment. This method can be used to compare digestion differences of a recombinant antibody between preterm and term infants. This approach can be used to establish the feasibility of using recombinant antibodies for enteric pathogen prevention in infants. We anticipate that this approach will be used to quantify digestion of a range of food proteins in infants and adults to assess the proteins that survive across the digestive tract.

## Figures and Tables

**Table 1:** List of tryptic peptides derived from palivizumab VL and VH segments.

Peptide Sequence ^a^	Modification	The Number of Detection of Each Peptide in Standards ^b^						Uniqueness ^c^
		2	5	10	15	20	25	
**Segment VL**								
APKLLIYDTSKLASGVPSR	Acetylation					++++	++++	Unique
DIQMTQSPSTLSASVGDR			+++	++++	++++	++++	++++	
LLIYDTSKLASGVPSR					+++	++++	++++	Unique
LLIYDTSK		++++	++++	++++	++++	++++	++++	Unique
LASGVPSR			+	+	+++	++	++	Unique
**Segment VH**								
VTNMDPADTATYYCAR	Carbamidomethylation	++++	++++	++++	++++	++++	++++	Unique
LTISKDTSKNQVVLK					++	++++	+++	Unique
KDYNPSLK	Acetylation		+++	++++	++++	++++	++++	Unique
SRLTISK	Phosphorylation			++++	++++	++++	++++	
NQVVLK				++	++++	++++	++++	

^a^ Peptides in VL and VH segments of palivizumab determined by full scanning using a nano LC/Orbitrap MS. ^b^ The number of peptides detected by four instrumental measurements monitored at six concentrations (ng/µL) of standards. One ‘+’ symbol indicates that the peptide has been measured one time for all four measurements. ^c^ The peptide uniqueness checker in the neXtProt (https://www.nextprot.org/) was used to determine uniqueness of peptides. A minimum of six amino acids were used to determine peptide uniqueness. DIQMTQSPSTLSASVGDR, SRLTISK and NQVVLK found from protein sequences of P01602, A0A0B4J1V2 and Q07020 (primary accession number of UniProt), respectively.

**Table 2:** Transitions used for PRM-based quantitation.

Unique Peptide ^a^ of Palivizumab		PEG-28	
Fragment Ions	m/z [M+H]^+^	Fragment Ions	m/z [M+H]^+^
y2	234.1448	DP ^c^ 7	327.2013
y3	335.1925	DP 8	371.2276
y4	450.2195	DP 10	459.2800
y5	613.2828	DP 11	503.3062
y6	726.3668	DP 12	547.3324
y7	839.4509	DP 13	591.3586
**SIS Peptide ^b^**		DP 14	635.3848
**Fragment Ions**	**m/z [M+H]^+^**	DP 15	679.4111
y2	242.1590	DP 16	723.4373
y3	343.2067	DP 17	767.4635
y4	458.2337		
y5	621.2970		
y6	734.3810		
y7	847.4651		

^a^ Peptide sequence is LLIYDTSK (m/z 476.771, z = 2). ^b^ K position of LLIYDTSK was labeled with ^13^C_6_ and ^15^N_2_ (m/z 480.778, z = 2). ^c^ Degree of polymer.

**Table 3:** Concentrations of palivizumab found from feed (human milk), gastric, intestinal and stool samples by parallel-reaction monitoring (PRM)-based quantitation.

Subject	Sample Type	Conc. (ng/µL) ^a^	Dilution Factor ^b^	Normalized Conc. (ng/µL) ^c^	Error ^d^	CV (%) ^e^
Infant 1	Feed	38.10	1	38.10	1.11	5.84
	Gastric	32.26	1.004	32.41	0.31	1.91
	Intestinal	10.04	1.14	11.45	0.20	3.57
	Stool	N/D	0.45	-	-	-
Infant 2	Feed	18.66	1	18.66	0.29	3.12
	Gastric	11.70	1.25	14.67	0.30	4.08
	Intestinal	1.63	1.51	2.47	0.01	1.01
	Stool	1.97	1.14	2.25	0.005	0.44
Infant 3	Feed	18.51	1	18.51	0.27	2.87
	Gastric	16.14	1.14	18.44	0.20	2.21
	Intestinal	5.48	1.30	7.14	0.08	2.34
	Stool	1.24	0.76	0.95	0.002	0.45
Infant 4	Feed	16.17	1	16.17	0.47	5.81
	Gastric	14.42	1.01	14.57	0.36	4.90
	Intestinal	5.39	1.14	6.16	0.09	2.82
	Stool	0.65	1.23	0.80	0.02	3.30

^a^ Palivizumab concentrations acquired from the four instrumental measurements were normalized based on the measured concentration of the stable isotope-labeled synthetic (SIS) peptide and then, these values were averaged. ^b^ Dilution factor = concentration of PEG-28 measured in the feed ÷ concentration of PEG-28 measured in the digestion sample. ^c^ Concentrations of palivizumab in gastric, intestinal and stool samples were normalized based on the dilution factor. ^d^ Error = standard deviation ÷ (number of measurements)^1/2^. ^e^ CV = (standard deviation ÷ average of normalized concentrations) × 100.

## Supplementary Materials

The following are available online at https://www.mdpi.com/2304-8158/9/6/759/s1. Figure S1: Extracted ion chromatograms (top) and tandem MS spectrum (bottom) of PEG-28 (m/z 417.922, z = 3) from the supernatant portion collected after ethanol precipitation. PEG-28 was analyzed by full MS/MS scanning (60 min) using C18-nano LC/Orbitrap MS. Figure S2: Tandem MS spectra of PEG-28 (m/z 417.922, z = 3) fragmented by HCD in C18-nano LC/Orbitrap MS. Figure S3: MS spectra of PEG polymers in PEG 3350 acquired from full MS scanning using C18-nano LC/Orbitrap MS. Table S1: Characteristics of four infants and the feed, gastric, intestinal and stool contents. Raw data for Table 3 and Figure 4.
