# Supplementary material for: Quantitative Analysis of Antibody Survival across the Infant Digestive Tract Using Mass Spectrometry with Parallel Reaction Monitoring
Source: Foods. 2020 Jun 9;9(6):759. doi: 10.3390/foods9060759 (PMC7353590; doi:10.3390/foods9060759)
Supplement: Supplementary file 1 [file foods-09-00759-s001.zip › Supplementary Figure Table for Foods (A revised version).pptx]

## Slide 1
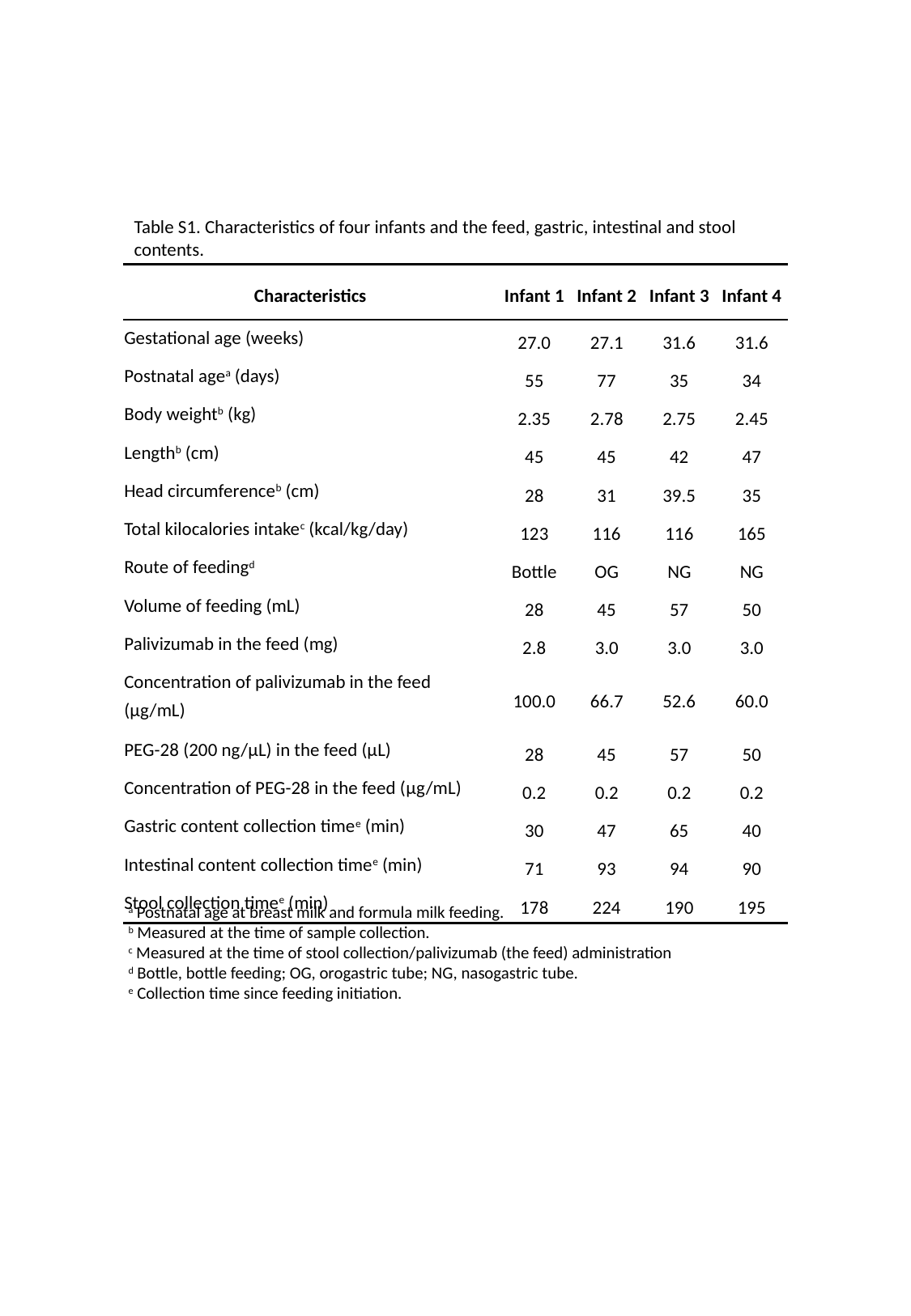

Table S1. Characteristics of four infants and the feed, gastric, intestinal and stool contents.
| Characteristics | Infant 1 | Infant 2 | Infant 3 | Infant 4 |
| --- | --- | --- | --- | --- |
| Gestational age (weeks) | 27.0 | 27.1 | 31.6 | 31.6 |
| Postnatal agea (days) | 55 | 77 | 35 | 34 |
| Body weightb (kg) | 2.35 | 2.78 | 2.75 | 2.45 |
| Lengthb (cm) | 45 | 45 | 42 | 47 |
| Head circumferenceb (cm) | 28 | 31 | 39.5 | 35 |
| Total kilocalories intakec (kcal/kg/day) | 123 | 116 | 116 | 165 |
| Route of feedingd | Bottle | OG | NG | NG |
| Volume of feeding (mL) | 28 | 45 | 57 | 50 |
| Palivizumab in the feed (mg) | 2.8 | 3.0 | 3.0 | 3.0 |
| Concentration of palivizumab in the feed (μg/mL) | 100.0 | 66.7 | 52.6 | 60.0 |
| PEG-28 (200 ng/μL) in the feed (μL) | 28 | 45 | 57 | 50 |
| Concentration of PEG-28 in the feed (μg/mL) | 0.2 | 0.2 | 0.2 | 0.2 |
| Gastric content collection timee (min) | 30 | 47 | 65 | 40 |
| Intestinal content collection timee (min) | 71 | 93 | 94 | 90 |
| Stool collection timee (min) | 178 | 224 | 190 | 195 |
a Postnatal age at breast milk and formula milk feeding.
b Measured at the time of sample collection.
c Measured at the time of stool collection/palivizumab (the feed) administration
d Bottle, bottle feeding; OG, orogastric tube; NG, nasogastric tube.
e Collection time since feeding initiation.

## Slide 2
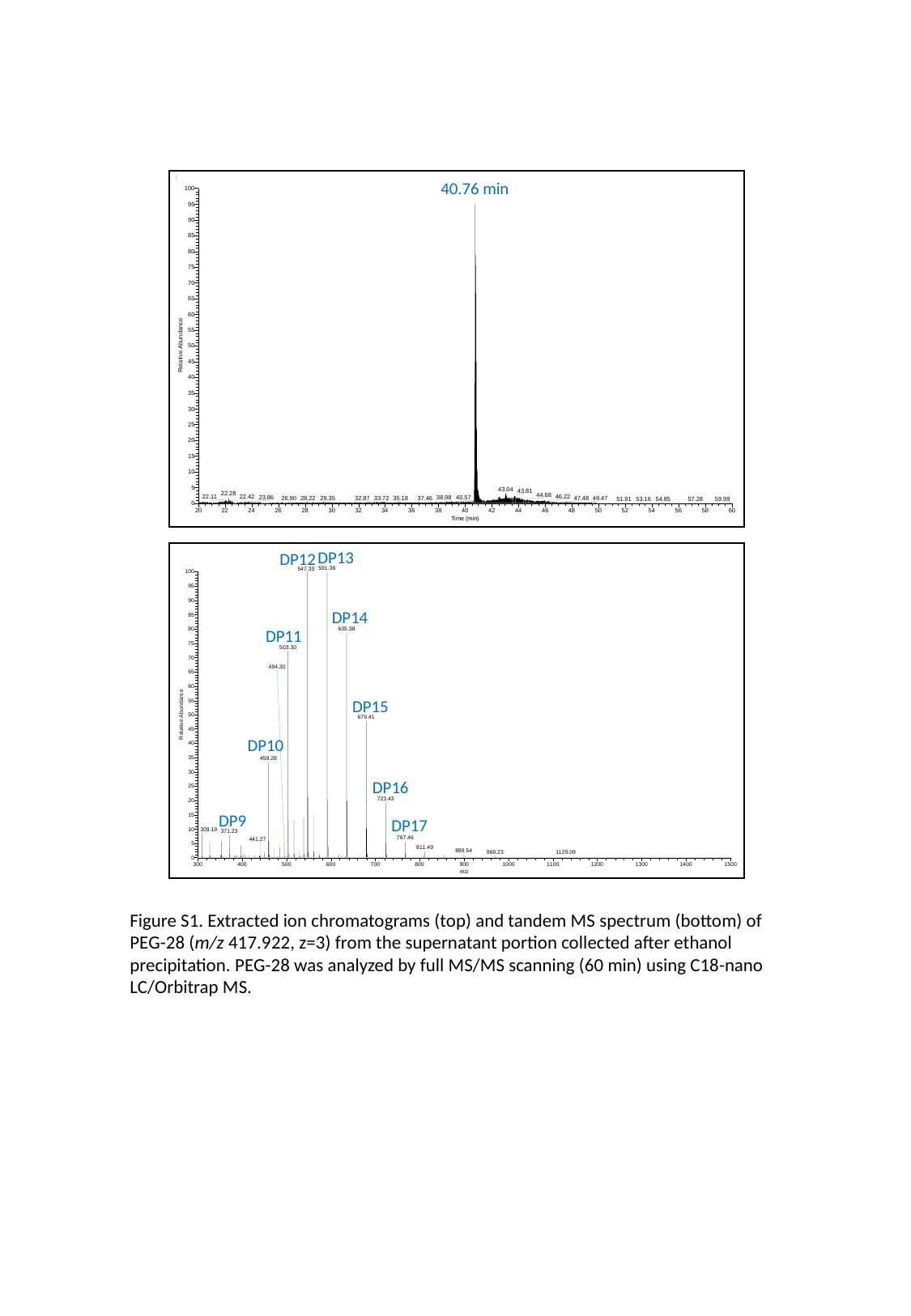

40.76 min
DP13
DP12
DP14
DP11
DP15
DP10
DP16
DP9
DP17
Figure S1. Extracted ion chromatograms (top) and tandem MS spectrum (bottom) of PEG-28 (m/z 417.922, z=3) from the supernatant portion collected after ethanol precipitation. PEG-28 was analyzed by full MS/MS scanning (60 min) using C18-nano LC/Orbitrap MS.

## Slide 3
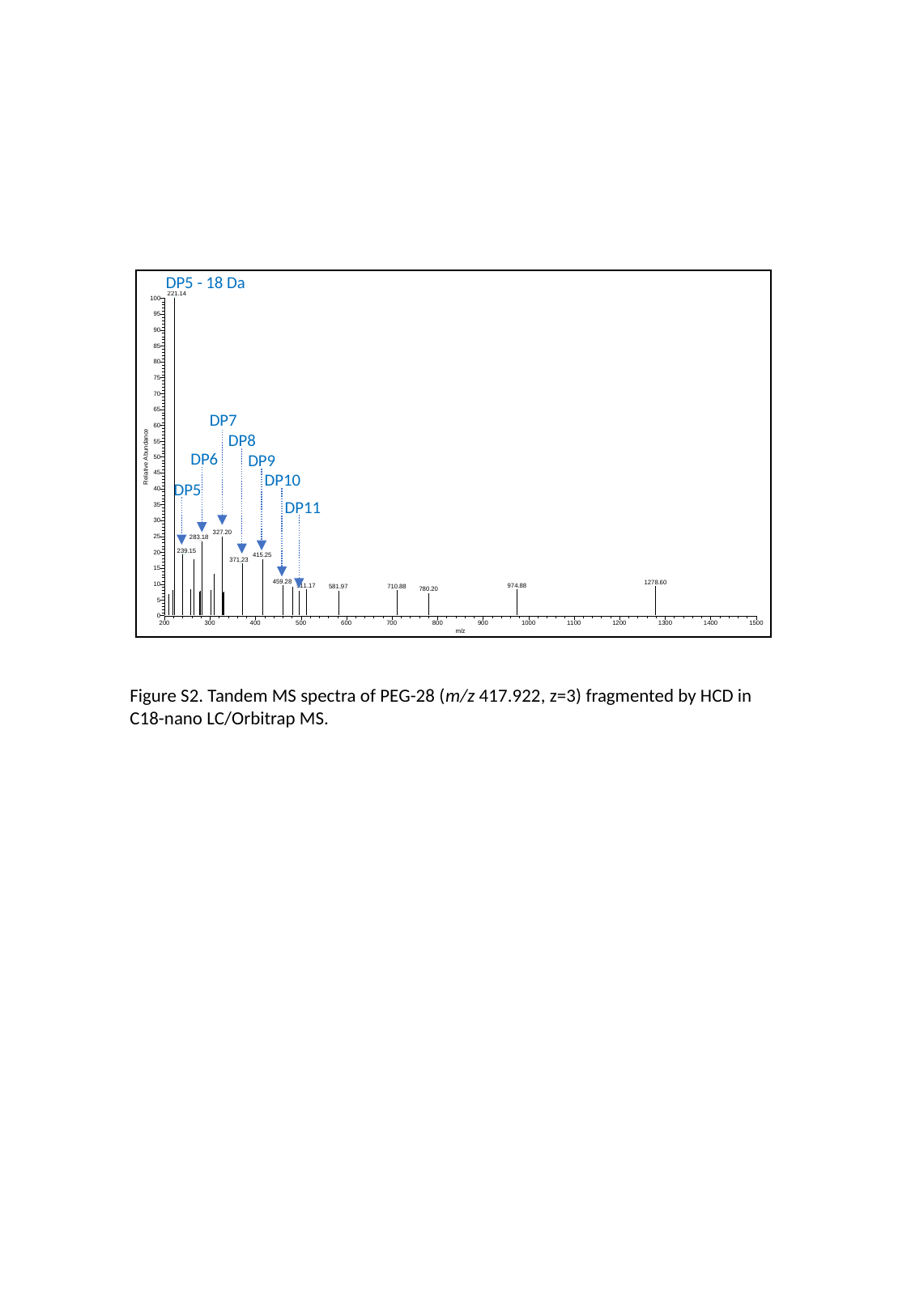

DP5 - 18 Da
DP7
DP8
DP6
DP9
DP10
DP5
DP11
Figure S2. Tandem MS spectra of PEG-28 (m/z 417.922, z=3) fragmented by HCD in C18-nano LC/Orbitrap MS.

## Slide 4
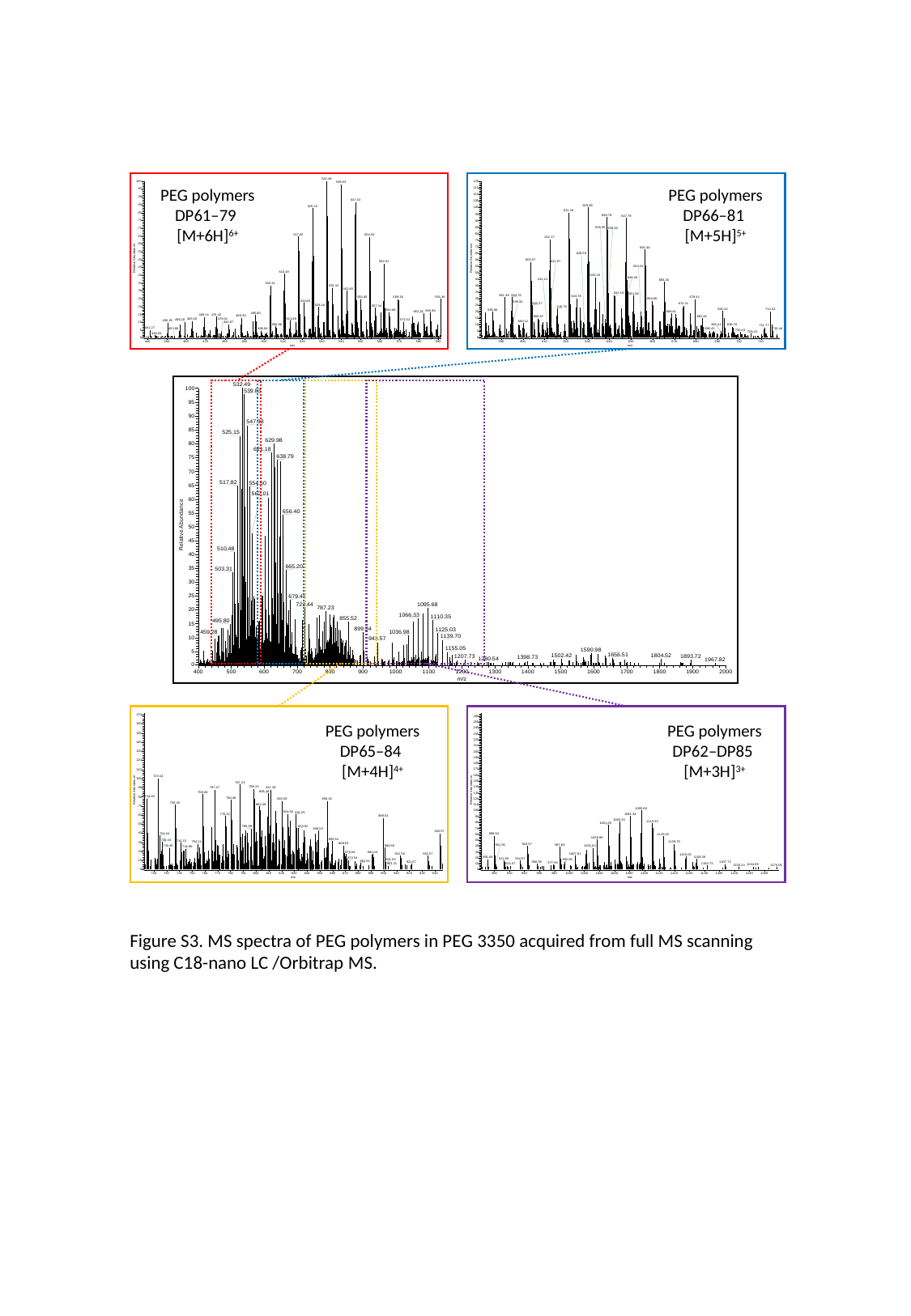

PEG polymers
DP61–79
[M+6H]6+
PEG polymers DP66–81
[M+5H]5+
PEG polymers
DP65–84
[M+4H]4+
PEG polymers DP62–DP85
[M+3H]3+
Figure S3. MS spectra of PEG polymers in PEG 3350 acquired from full MS scanning using C18-nano LC /Orbitrap MS.
